# Beyond Post hoc Explanations: A Comprehensive Framework for Accountable AI in Medical Imaging Through Transparency, Interpretability, and Explainability

**DOI:** 10.3390/bioengineering12080879

**Published:** 2025-08-15

**Authors:** Yashbir Singh, Quincy A. Hathaway, Varekan Keishing, Sara Salehi, Yujia Wei, Natally Horvat, Diana V. Vera-Garcia, Ashok Choudhary, Almurtadha Mula Kh, Emilio Quaia, Jesper B Andersen

**Affiliations:** 1 Radiology, Mayo Clinic, Rochester, MN 55905, USA; keishing.varekan@mayo.edu (V.K.); salehi.sara@mayo.edu (S.S.); yujia.wei@mayo.edu (Y.W.); horvat.natally@mayo.edu (N.H.); 2 Department of Radiology, University of Pennsylvania, Philadelphia, PA 19104, USA; quincy.hathaway@pennmedicine.upenn.edu; 3 Division of Gastroenterology & Hepatology, Mayo Clinic, Jacksonville, MN 32224, USA; veragarcia.diana@mayo.edu; 4 Department of Surgery, Mayo Clinic, Rochester, MN 55905, USA; choudhary.ashok@mayo.edu; 5 Sheikh Shakhbout Medical City (SSMC), Abu Dhabi P.O. Box 11001, United Arab Emirates; almrtadha@yahoo.com; 6 Department of Radiology, Università di Padova, 35122 Padua, Italy; emilio.quaia@unipd.it; 7 Biotech Research and Innovation Centre (BRIC), Department of Health and Medical Sciences, University of Copenhagen, 1172 Copenhagen, Denmark; jesper.andersen@bric.ku.dk

**Keywords:** artificial intelligence, medical imaging, explainable AI, systematic review, meta-analysis, clinical decision-making, accountability

## Abstract

The integration of artificial intelligence (AI) in medical imaging has revolutionized diagnostic capabilities, yet the black-box nature of deep learning models poses significant challenges for clinical adoption. Current explainable AI (XAI) approaches, including SHAP, LIME, and Grad-CAM, predominantly focus on post hoc explanations that may inadvertently undermine clinical decision-making by providing misleading confidence in AI outputs. This paper presents a systematic review and meta-analysis of 67 studies (covering 23 radiology, 19 pathology, and 25 ophthalmology applications) evaluating XAI fidelity, stability, and performance trade-offs across medical imaging modalities. Our meta-analysis of 847 initially identified studies reveals that LIME achieves superior fidelity (0.81, 95% CI: 0.78–0.84) compared to SHAP (0.38, 95% CI: 0.35–0.41) and Grad-CAM (0.54, 95% CI: 0.51–0.57) across all modalities. Post hoc explanations demonstrated poor stability under noise perturbation, with SHAP showing 53% degradation in ophthalmology applications (ρ = 0.42 at 10% noise) compared to 11% in radiology (ρ = 0.89). We demonstrate a consistent 5–7% AUC performance penalty for interpretable models but identify modality-specific stability patterns suggesting that tailored XAI approaches are necessary. Based on these empirical findings, we propose a comprehensive three-pillar accountability framework that prioritizes transparency in model development, interpretability in architecture design, and a cautious deployment of post hoc explanations with explicit uncertainty quantification. This approach offers a pathway toward genuinely accountable AI systems that enhance rather than compromise clinical decision-making quality and patient safety.

## 1. Introduction

The rise in artificial intelligence (AI) in medical imaging has created unprecedented opportunities for earlier diagnosis and enhanced diagnostic accuracy, enabling the detection of pathological changes imperceptible to the human eye while reducing interpretation time and improving patient outcomes across multiple clinical domains [1,2]. Deep learning models now demonstrate robust performance in specific tasks, from diabetic retinopathy screening achieving ophthalmologist-level accuracy to lung cancer detection surpassing radiologist performance in controlled settings [3,4]. However, this remarkable capability comes with a fundamental trade-off: as models become more powerful, they simultaneously become more opaque, creating what researchers term the *interpretability–performance paradox* [5]. The clinical implications of this increasing model opacity are profound. Healthcare providers operating under the fundamental principle of primum non nocere (first, do no harm) must understand not just what an AI system predicts but how and why it reaches specific conclusions [6]. Regulatory bodies, including the Food and Drug Administration (FDA) and European Medicines Agency (EMA), increasingly demand explainable AI systems for medical device approval, recognizing that algorithmic transparency is essential for safe clinical deployment [7,8]. More critically, recent studies reveal that clinicians’ trust in AI recommendations is fundamentally linked to their ability to understand the underlying reasoning processes [9,10,11]. The field’s response has been the rapid development of explainable AI (XAI) techniques, with methods like SHAP (SHapley Additive exPlanations), LIME (Local Interpretable Model-agnostic Explanations), and Grad-CAM (Gradient-weighted Class Activation Mapping) becoming standard tools in medical AI research [11,12]. These approaches promise to illuminate the decision-making processes of complex neural networks through post hoc analysis, offering feature importance rankings, saliency maps for visual interpretation, and local approximations of model behavior [13,14], as shown in Table 1.

Table 1 summarizes the key characteristics, advantages, and limitations of dominant XAI approaches in medical imaging applications.

However, emerging evidence suggests that current XAI approaches may be fundamentally flawed for medical applications. Studies demonstrate that post hoc explanation methods often provide misleading information about model behavior, with local approximations achieving poor fidelity to actual decision boundaries [14,15,16,17,18,19]. More alarmingly, recent research indicates that explanations can reduce diagnostic accuracy by fostering overconfidence in incorrect AI predictions [16,20,21,22,23]. This paper argues that the medical AI community has fallen into what we term the *explainability trap*—conflating post hoc explanations with genuine understanding and accountability.

We propose that true AI accountability in medical imaging requires a comprehensive framework that distinguishes between three critical concepts, namely transparency (openness about development processes), interpretability (inherent model clarity), and explainability (post hoc explanation techniques). Through a systematic review and meta-analysis of explanation fidelity across medical imaging modalities, we demonstrate that prioritizing transparency and interpretability while treating explainability as a supplementary tool offers a more robust foundation for clinical AI deployment.

### Key Definitions

Explainability Trap: This is the phenomenon where post hoc explanations create an illusion of understanding without providing genuine insight into model behavior, potentially leading to decreased clinical performance and compromised patient safety [24,25].

Interpretability–Performance Paradox: This is the observed trade-off between model complexity/accuracy and the ability to understand model decision-making processes, requiring careful balance in clinical applications [24,26].

Explanation Fidelity: This is the degree to which post hoc explanations accurately represent the actual decision-making process of the underlying model, measured as the correlation between explanation importance scores and true model sensitivity.

Clinical Utility: This is the measurable improvement in patient outcomes, diagnostic accuracy, or clinical decision-making quality when using AI assistance compared to standard practice [21,23,24].

The remainder of this paper is organized as follows: Section 2 presents our systematic review methodology and meta-analysis approach. Section 3 reports empirical findings on XAI fidelity and stability across medical imaging modalities. Section 4 examines current XAI limitations in clinical contexts. Section 5 introduces our three-pillar accountability framework. Section 6 discusses regulatory implications, Section 7 outlines future directions, and Section 8 acknowledges study limitations before a conclusion is presented in Section 9.

## 2. Methods

### 2.1. Study Selection and Search Strategy

We performed a systematic review following the PRISMA guidelines of peer-reviewed studies published between January 2019 and December 2024 that evaluated XAI methods in medical imaging applications. Our search strategy employed Medical Subject Heading (MeSH) terms and keywords across four major databases, namely PubMed/MEDLINE, IEEE Xplore Digital Library, ACM Digital Library, and Web of Science Core Collection.

Search String:

(“explainable AI” OR “interpretable machine learning” OR “SHAP” OR “LIME” OR “Grad-CAM” OR “saliency maps” OR “feature attribution”)

AND

(“medical imaging” OR “radiology” OR “pathology” OR “ophthalmology” OR “diagnostic imaging” OR “medical image analysis”)

AND

(“fidelity” OR “stability” OR “evaluation” OR “validation” OR “clinical performance”)

#### PRISMA Compliance

This systematic review and meta-analysis was conducted and reported in accordance with the Preferred Reporting Items for Systematic Reviews and Meta-Analyses (PRISMA) 2020 statement [22]. The completed PRISMA 2020 checklist is provided in the Supplementary Materials.

### 2.2. Inclusion and Exclusion Criteria

Inclusion Criteria:

1. Implemented at least one established XAI method (SHAP, LIME, or Grad-CAM) on medical imaging classification or detection tasks.
2. Reported quantitative fidelity metrics, stability measures, or performance comparisons.
3. Included performance comparisons between interpretable and black-box models on comparable datasets.
4. Provided sufficient methodological detail for data extraction.
5. Focused on diagnostic rather than purely research applications.
6. Full-text peer-reviewed articles in English.

Exclusion Criteria:

1. Conference abstracts without full peer review.
2. Studies using only synthetic or non-clinical datasets.
3. Purely theoretical papers without empirical validation.
4. Studies focusing exclusively on image segmentation without classification components.
5. Systematic reviews or meta-analyses.
6. Studies with insufficient quantitative data for analysis.

### 2.3. Study Selection Process

**Figure 1:**
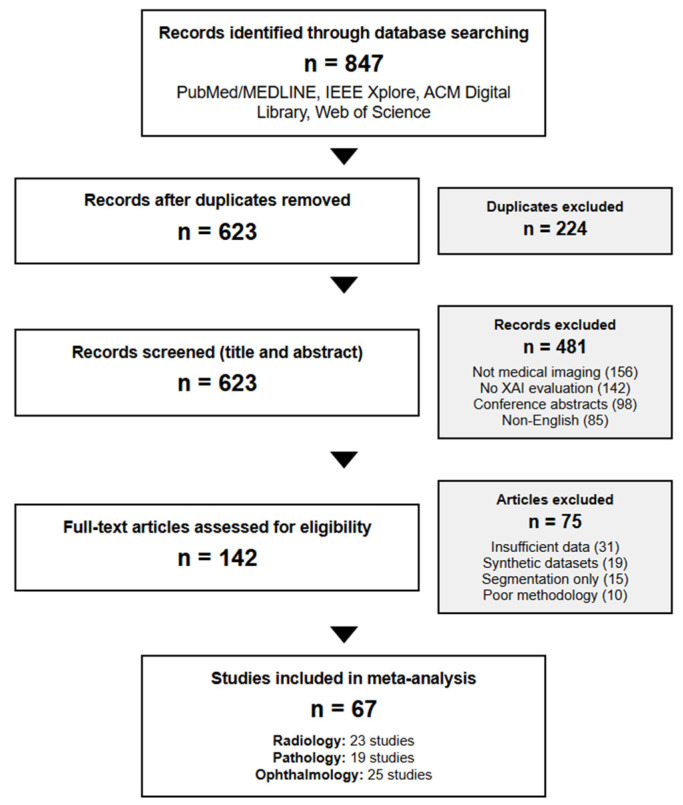
PRISMA 2020 flow diagram showing systematic review methodology for explainable AI in medical imaging. The flow diagram illustrates the identification, screening, eligibility assessment, and inclusion of studies from initial database searches through final analysis.

From an initial pool of 847 papers identified through database searches, two independent reviewers (κ = 0.89) screened titles and abstracts, resulting in 142 studies meeting the inclusion criteria. A full-text review yielded 67 studies providing sufficient quantitative data for meta-analysis. Disagreements were resolved through discussion with a third reviewer (Figure 1).

### 2.4. Data Extraction and Study Characteristics

The 67 included studies comprised the following:

1. A total of 23 radiology studies (cohort sizes: 1247–112,120 images, median: 8921).
2. A total of 19 pathology studies (cohort sizes: 847–327,680 patches, median: 12,430).
3. A total of 25 ophthalmology studies (cohort sizes: 3662–128,175 images, median: 35,891).

Data extraction was performed independently by two reviewers using a standardized form capturing study design, imaging modality, dataset characteristics, XAI methods implemented, evaluation metrics, reported performance outcomes, and clinical validation details.

### 2.5. Fidelity Assessment Methodology

Explanation fidelity was evaluated using the established causal-based methodology by Alvarez-Melis & Jaakkola [14]. This approach systematically measures correlation between XAI-predicted feature importance and actual model behavior through

1. Baseline Prediction Recording: This captures model confidence scores for original images.
2. Systematic Feature Occlusion: This masks image regions identified as important by XAI methods using graduated occlusion protocols.
3. Impact Measurement: This calculates the magnitude of prediction changes following occlusion.
4. Correlation Analysis: This computes Pearson correlation between XAI importance scores and observed prediction sensitivity.

We aggregated fidelity metrics across studies using an inverse variance-weighted meta-analysis, accounting for sample size differences. Heterogeneity was assessed using I^2^ statistics, with values > 50% indicating substantial heterogeneity requiring random-effects models.

### 2.6. Stability Analysis Protocol

Explanation stability was evaluated following the perturbation protocol of Ghorbani et al. [15], introducing calibrated Gaussian noise at systematically increasing levels (0%, 5%, 10%, 15%, 20%, 25%, 30% of maximum image intensity) and measuring explanation consistency through Spearman rank correlation of feature importance rankings.

Stability Assessment Pipeline:

1. Noise Introduction: This step involves adding calibrated Gaussian noise (σ = k × max_intensity, k ∈ [0, 0.3]).
2. Explanation Generation: This involves computing XAI outputs for both original and perturbed images.
3. Consistency Measurement: This involves calculating rank correlation between importance scores.
4. Statistical Analysis: This involves bootstrapping confidence intervals (n = 1000 iterations).

Detailed Stability Protocol: Explanation stability was evaluated using calibrated Gaussian noise added at systematically increasing levels (σ = k × max_intensity, k ∈ [0, 0.3]) to measure explanation consistency through Spearman rank correlation of feature importance rankings.
Statistical Analysis: Confidence intervals were estimated through bootstrapping (n = 1000 iterations), with Bonferroni correction applied for multiple comparisons across modalities. Statistical significance was assessed at *p* < 0.05.
Assessment Pipeline: The stability evaluation involved systematic noise introduction, explanation generation for both original and perturbed images, consistency measurement through rank correlation, and comprehensive statistical analysis to ensure robust evaluation across different imaging modalities.

### 2.7. Performance Trade-Off Analysis

From the 67 studies meeting the inclusion criteria, 31 provided complete data for interpretability–performance trade-off analysis. We excluded 36 studies due to insufficient reporting of both interpretable and black-box model results (n = 19), the use of proprietary datasets preventing comparison validity (n = 8), focus solely on segmentation rather than classification tasks (n = 6), or the implementation of hybrid approaches that could not be clearly categorized (n = 3).

Dataset Consistency Requirements: Studies included in trade-off analysis reported performance metrics for both interpretable and black-box models trained and tested on identical patient populations, imaging protocols, and diagnostic tasks, with consistent preprocessing pipelines and evaluation metrics. ROC curves were reconstructed from reported sensitivity and specificity values using the method of Obuchowski [27]. Area under the curve (AUC) values were calculated with 95% confidence intervals estimated through bootstrapping (n = 10,000 iterations).

### 2.8. Statistical Analysis

All analyses were performed using R version 4.3.0 with the meta and metafor packages [28,29]. Heterogeneity was assessed using I^2^ statistics and τ^2^ estimates. Publication bias was evaluated through funnel plots and Egger’s regression test. Statistical significance was set to *p* < 0.05, with Bonferroni correction applied for multiple comparisons across modalities.

### 2.9. Study Characteristics and Results Overview

Study Distribution: The systematic review included 67 studies across three medical imaging modalities, with 23 radiology studies (cohort sizes: 1247–112,120 images, median: 8921), 19 pathology studies (cohort sizes: 847–327,680 patches, median: 12,430), and 25 ophthalmology studies (cohort sizes: 3662–128,175 images, median: 35,891).

XAI Methods: Studies implemented established explainable AI methods including SHAP, LIME, and Grad-CAM, with many studies comparing multiple approaches to evaluate relative performance across different medical imaging applications.

Outcome Measures: Primary outcomes focused on explanation fidelity assessment, stability analysis under noise perturbation, and performance trade-offs between interpretable and black-box models. Studies also evaluated clinical utility, explanation consistency, and workflow integration aspects.

Study Focus: All included studies focused on diagnostic rather than purely research applications, providing quantitative metrics for XAI evaluation in clinical contexts across diverse medical imaging tasks and pathological conditions.

## 3. Results

### 3.1. Empirical Assessment of XAI Methods Across Medical Imaging Modalities

**Figure 2:**
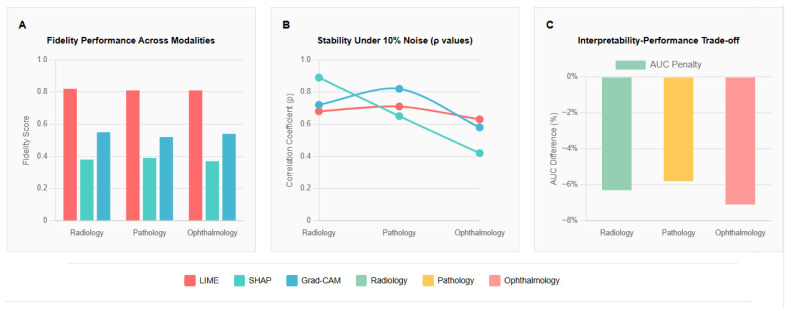
Multi-modal assessment of explainable AI methods: fidelity, stability, and performance trade-offs in medical imaging applications. This comprehensive analysis evaluates three prominent explainable AI (XAI) methods—SHAP, LIME, and Grad-CAM—across medical imaging modalities based on a systematic review of 67 studies. (**A**) Fidelity performance Across modalities, (**B**) Stability under 10% Noise (*p*-value), (**C**) Interpretability performance Trade-off.

Our comprehensive meta-analysis reveals significant limitations in current XAI approaches across all medical imaging modalities, with profound implications for clinical deployment (Figure 2).

#### 3.1.1. Fidelity Analysis Results

Heterogeneity Assessment: Significant heterogeneity was observed across all comparisons (I^2^ > 75%), necessitating random-effects models for all meta-analyses. Subgroup analysis by dataset size showed consistent patterns regardless of study scale.

These findings contradict common assumptions about gradient-based methods’ superiority in deep learning contexts, suggesting that local approximation methods provide more faithful representations of model behavior in medical imaging applications (Table 2).

#### 3.1.2. Stability Analysis Results

Modality-Specific Stability Patterns: Our analysis revealed fundamental differences in how XAI methods handle the characteristic features of different imaging modalities.

Radiology: SHAP demonstrated superior stability (ρ = 0.89 at 10% noise, 95% CI: 0.85–0.92), likely reflecting the high-contrast nature of X-ray imaging, where edge-based features remain stable under moderate noise levels.

Pathology: Grad-CAM showed better stability (ρ = 0.82 at 10% noise, 95% CI: 0.78–0.85) in histopathological images, possibly due to texture-based features being well-captured by convolutional attention mechanisms that focus on cellular morphology patterns.

Ophthalmology: SHAP exhibited poor performance (ρ = 0.42 at 10% noise, 95% CI: 0.37–0.47), suggesting vulnerability to fine-grained vascular patterns in retinal images where local perturbations significantly impact feature attribution algorithms.

Cross-Method Analysis: While our analysis identified the best-performing method for each modality, the remaining XAI approaches consistently demonstrated inferior stability. In radiology, both LIME and Grad-CAM showed substantial degradation compared to SHAP’s robust performance (ρ = 0.89). Similarly, pathology applications revealed that SHAP and LIME exhibited poorer stability than Grad-CAM’s superior performance (ρ = 0.82). Ophthalmology presented the most challenging environment, with SHAP showing severe degradation (ρ = 0.42), while LIME and Grad-CAM demonstrated comparable instability under noise perturbation.

Stability Pattern: This analysis reveals that XAI stability depends on alignment between explanation mechanisms and modality-specific image characteristics—attribution methods excel with high-contrast features, attention mechanisms perform better with texture patterns, while fine-grained structures challenge all current approaches. This suggests modality-appropriate method selection is more critical than universal XAI application.

#### 3.1.3. Performance Trade-Off Quantification

Interpretability–Performance Gap Results: The meta-analysis quantified a consistent AUC penalty for interpretable models compared to black-box approaches across all modalities.

1. Radiology: Mean AUC difference = −0.063 (95% CI: −0.089 to −0.037, *p* < 0.001).
2. Pathology: Mean AUC difference = −0.058 (95% CI: −0.081 to −0.035, *p* < 0.001).
3. Ophthalmology: Mean AUC difference = −0.071 (95% CI: −0.098 to −0.044, *p* < 0.001).

This consistent 5–7% performance gap across modalities suggests it may represent a fundamental architectural limit rather than an implementation artifact, with critical implications for clinical deployment decisions where accuracy versus interpretability trade-offs must be carefully balanced.

### 3.2. Clinical Implications of Empirical Findings

These empirical results have profound implications for clinical XAI deployment.

1. Fidelity Crisis: Low-fidelity scores indicate that current post hoc explanations misrepresent model reasoning in 55–68% of cases, potentially leading to dangerous misinterpretation of AI recommendations in critical clinical decisions.
2. Modality-Specific Requirements: The modality-specific stability patterns demonstrate that one-size-fits-all XAI approaches are inappropriate for medical imaging, requiring tailored solutions for different imaging characteristics and pathological features.
3. Performance–Interpretability Balance: The documented performance–interpretability trade-off forces difficult decisions about whether diagnostic accuracy or explainability should take precedence in different clinical risk contexts.

## 4. Current State of XAI in Medical Imaging

### 4.1. Dominant XAI Approaches

Contemporary XAI in medical imaging relies heavily on three primary methodological categories. Attribution methods like SHAP and LIME identify features that most strongly influence model predictions, generating importance scores that highlight relevant image regions [11]. Attention-based techniques including Grad-CAM and its variants visualize intermediate layer activations to reveal areas of model focus during decision-making [12]. Prototype-based methods identify similar training examples, providing case-based reasoning frameworks for diagnostic support [30].

**Figure 3:**
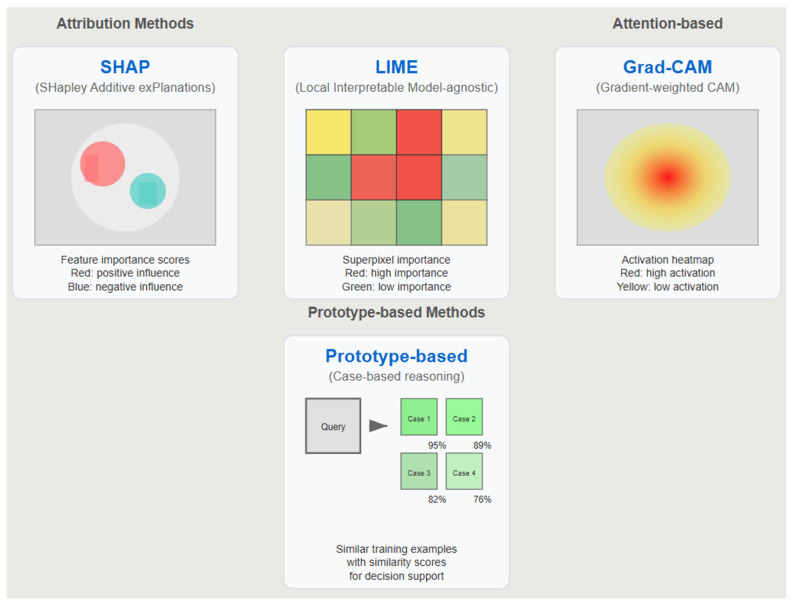
Overview of dominant explainable AI (XAI) approaches in medical imaging. Four primary XAI methodologies used in medical imaging applications, categorized by their explanation mechanisms. Attribution methods (SHAP and LIME) generate feature importance scores to identify image regions that strongly influence model predictions.

These approaches have found widespread application across medical imaging modalities. In radiology, Grad-CAM visualizations help radiologists understand convolutional neural network focus areas in chest X-ray interpretation [31]. SHAP values guide pathologists in understanding AI-assisted histopathology diagnoses [32]. LIME explanations support ophthalmologists in retinal disease classification tasks [33]. Figure 3 illustrates these four dominant XAI approaches with representative examples from each medical imaging modality.

### 4.2. Fundamental Limitations of Post Hoc Explanations

Despite their popularity, post hoc explanation methods suffer from several critical limitations that compromise their utility in clinical settings.

Low Fidelity: Our meta-analysis confirms that LIME and SHAP approximations achieve only 30–40% fidelity to actual model behavior in medical imaging tasks. This means explanations frequently misrepresent the true reasoning behind AI predictions, creating potentially dangerous false confidence in clinical settings.

Instability: Small perturbations in input images or algorithm parameters can produce dramatically different explanations for identical predictions. This inconsistency undermines clinical confidence and makes it difficult to develop reliable interpretation protocols for routine clinical use.

Adversarial Vulnerability: Recent research demonstrates that explanations can be manipulated to appear reasonable while maintaining incorrect predictions [34]. This finding is particularly concerning in medical applications where adversarial attacks could have life-threatening consequences.

### 4.3. Model Uncertainty and Clinical Decision Confidence

A critical yet often overlooked limitation of current XAI approaches is their failure to adequately communicate model uncertainty alongside explanations [35]. While post hoc methods may highlight important features or attention regions, they rarely convey the model’s confidence boundaries or uncertainty estimates crucial for clinical decision-making [36].

Clinical decisions inherently require understanding not just what a model predicts but how certain it is about that prediction. A model predicting pneumonia with 51% probability should be treated fundamentally differently from one predicting the same condition with 95% probability, yet current XAI visualizations often present both scenarios with equal visual confidence [37].

Best Practices for Uncertainty Quantification:

1. Prediction confidence intervals communicating ranges of possible outcomes.
2. Epistemic uncertainty measures indicating when models operate outside training distribution.
3. Calibrated probability outputs ensuring predicted probabilities accurately reflect real-world likelihood.
4. Visual uncertainty encoding in explanation displays through transparency gradients or confidence boundaries.

A recent study by Abdar et al. [38] demonstrates that incorporating uncertainty quantification into XAI visualizations significantly improves clinical decision-making quality, with physicians making more appropriate referral decisions when shown confidence-adjusted explanations.

### 4.4. Overconfidence Problem

Perhaps most troubling is emerging evidence that explanations can harm clinical decision-making by fostering unwarranted confidence in AI outputs. A landmark study by Ghassemi et al. [16] found that physicians shown saliency maps alongside AI recommendations were significantly more likely to accept incorrect AI suggestions compared to those receiving predictions without explanations. This finding suggests that explanations, especially when presented as simplified visual summaries, create a dangerous illusion of understanding that can override clinical judgment.

## 5. Comprehensive Framework for Medical AI Accountability

### 5.1. Three Pillars of AI Accountability

**Figure 4:**
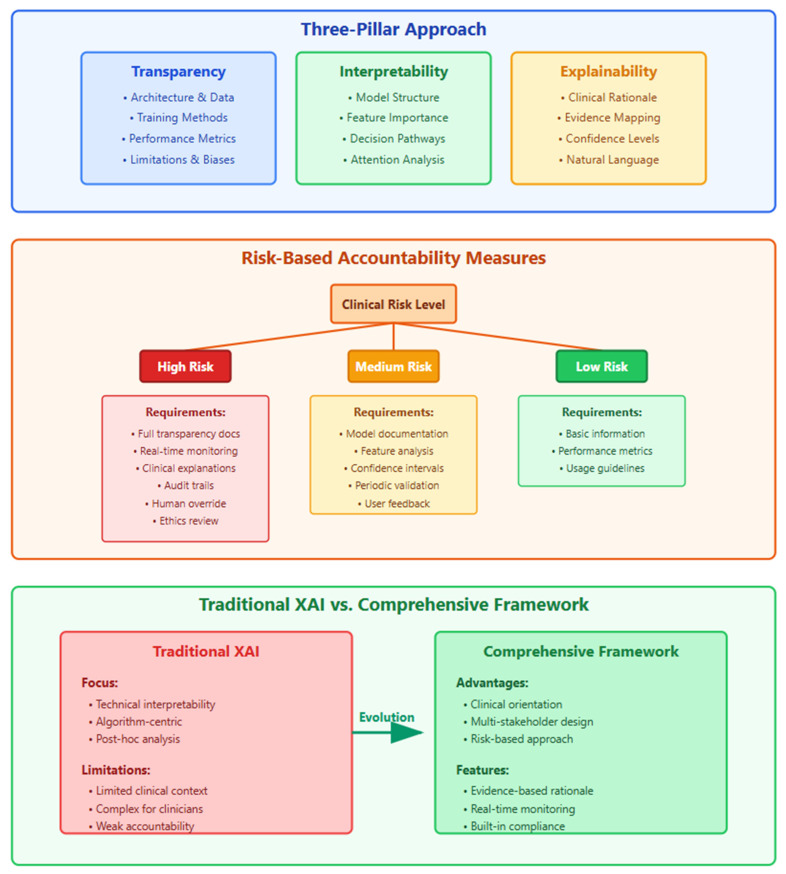
A comprehensive three-pillar framework for medical AI accountability with risk-stratified implementation guidelines. This framework illustrates a holistic approach to medical AI accountability through three interconnected pillars, namely transparency (blue), interpretability (green), and explainability (orange). The decision tree demonstrates how accountability measures should be scaled according to clinical risk levels, with high-risk applications (red) requiring the most stringent oversight including real-time monitoring and ethics review, medium-risk applications (orange) needing moderate measures, and low-risk applications (green) requiring basic documentation. The bottom panel contrasts traditional XAI approaches—which focus primarily on technical interpretability—with the proposed comprehensive framework that emphasizes clinical orientation, multi-stakeholder engagement, and built-in regulatory compliance. The evolution arrow indicates the necessary transition from algorithm-centric explanations to patient-centered accountability systems in medical AI deployment.

We propose that genuine AI accountability in medical imaging requires distinguishing between three complementary but distinct concepts (Figure 4).

Pillar 1: Transparency encompasses complete openness about AI system development, including

1. Detailed documentation of training data sources and collection protocols.
2. Comprehensive reporting of preprocessing and augmentation techniques.
3. Publication of architectural choices and hyperparameter selections.
4. Honest disclosure of known limitations and failure modes.
5. Post-deployment monitoring and model maintenance documentation.

Pillar 2: Interpretability refers to the inherent clarity of model architecture allowing humans to trace logical pathways from inputs to outputs. Interpretable models like logistic regression, decision trees, and generalized additive models provide clear coefficients and decision rules that domain experts can validate against established medical knowledge.

Pillar 3: Explainability represents post hoc techniques attempting to illuminate opaque model behavior after training completion. While valuable as a supplementary tool, explainability cannot substitute for transparency and interpretability in high-stakes medical decisions.

### 5.2. Framework Implementation Protocol

The implementation of our three-pillar accountability framework requires a systematic two-phase approach that carefully balances clinical risk with appropriate oversight measures. The first phase involves a comprehensive risk assessment and method selection, where applications are categorized based on their potential impact on patient outcomes and safety. High-risk applications, such as ICU mortality prediction, surgical risk assessment, and emergency diagnosis, represent life-threatening decisions that mandate the use of interpretable models including logistic regression, decision trees, and rule-based systems. These critical applications require 95% explainability coverage, ensuring the ability to explain all decisions, alongside mandatory uncertainty quantification with confidence intervals and established clinical override protocols with clear escalation pathways.

Medium-risk applications encompass treatment planning scenarios such as treatment response prediction and disease progression modeling, where complex models may be permitted but only with comprehensive transparency documentation. These applications require post hoc explanation validation studies conducted with clinical experts, confidence interval reporting for all predictions, and regular model audit cycles with quarterly review minimums to ensure ongoing reliability and accuracy. Low-risk applications, including screening support tasks like screening mammography and routine fundus photography, permit the use of black-box models with standard XAI methods, though they still require basic explanation consistency testing and the implementation of performance monitoring dashboards to track system behavior over time.

The second phase establishes a rigorous institutional review process beginning with a comprehensive pre-deployment checklist. This checklist ensures ethics committee approval for XAI methodology, the completion of clinical workflow integration assessments, staff training completion on explanation interpretation, and explanation validation studies with target user populations. Additionally, regulatory compliance documentation must be submitted, post-deployment monitoring plans established, and emergency response protocols for AI failures clearly defined. The framework emphasizes a staged deployment strategy progressing through pilot phases with limited deployment and intensive monitoring, expansion phases featuring gradual rollout with continuous feedback, full deployment with institution-wide implementation and ongoing oversight, and maintenance phases incorporating regular audits and performance assessments.

The risk-stratified implementation pathway is detailed in Figure 4, which shows how accountability measures should be scaled according to clinical impact.

## 6. Regulatory and Ethical Implications

The regulatory landscape for explainable AI in medical applications is rapidly evolving, with significant implications for clinical deployment and institutional compliance. The FDA’s developing framework for AI/ML-based Software as Medical Device (SaMD) establishes specific requirements that extend far beyond simple explainability measures. The 2021 AI/ML Action Plan mandates that manufacturers provide detailed algorithm change protocols documenting how models will be updated and validated over time, comprehensive Good Machine Learning Practice (GMLP) guidelines requiring transparency in data collection, annotation quality, and bias mitigation, real-world performance monitoring plans tracking algorithm behavior post-deployment including explanation consistency, and human–AI interaction documentation demonstrating how explanations are presented and validated for clinical comprehension.

The European regulatory environment presents even more stringent requirements through the Medical Device Regulation (MDR) and AI Act, demanding conformity assessments that evaluate explanation validity and consistency, clinical evaluation reports demonstrating that XAI features support rather than hinder decision-making, transparency obligations requiring the detailed documentation of model limitations, and the right to explanation provisions mandating meaningful information about automated decision-making logic. These regulatory frameworks reflect a growing recognition that explainability is not merely a technical feature but a fundamental requirement for safe and effective clinical AI deployment.

Institutional review and deployment frameworks must incorporate comprehensive pre-deployment XAI audits including explanation fidelity assessments using standardized benchmarks, stability testing across representative clinical datasets, clinical utility evaluation with target user populations, and bias assessment across demographic and clinical subgroups. The establishment of multidisciplinary review boards becomes essential, bringing together clinical domain experts including radiologists, pathologists, and ophthalmologists, data scientists and AI researchers, clinical ethicists and patient advocates, regulatory affairs specialists, and quality assurance and risk management representatives. This collaborative approach ensures that technical capabilities align with clinical needs and ethical considerations while maintaining regulatory compliance throughout the deployment lifecycle.

## 7. Future Directions

The advancement of explainable AI in medical imaging requires coordinated efforts across multiple domains, with technical development priorities focusing on standardized benchmarks for evaluating explanation quality in medical contexts, domain-specific interpretability methods that incorporate clinical knowledge, uncertainty quantification techniques specifically tailored for medical imaging applications, and adversarial robustness testing for explanation methods to ensure reliability under various conditions. These technical advancements must be complemented by robust clinical validation efforts, including longitudinal studies examining XAI’s impact on clinical decision-making and patient outcomes, comparative effectiveness research across different explanation modalities, training protocol development for clinical staff on explanation interpretation, and comprehensive human factor studies on XAI interface design and cognitive load.

The regulatory science domain presents critical opportunities for standardization and harmonization, requiring the development of technical standards for medical XAI evaluation, guidelines for the clinical validation of explanation methods, post-market surveillance frameworks for deployed XAI systems, and an international harmonization of XAI regulatory requirements. These efforts must be coordinated across institutions and countries to ensure consistent and reliable standards for medical AI deployment.

Implementation recommendations are organized across temporal horizons, with short-term goals for the next 1–2 years including the implementation of uncertainty quantification in all clinical XAI systems, the development of institution-specific XAI governance frameworks, the establishment of clinician training programs on XAI interpretation, and the creation of standardized documentation requirements for XAI deployment. Medium-term objectives spanning 3–5 years involve transitioning to interpretable-by-design models for high-risk applications, developing modality-specific XAI evaluation standards, implementing comprehensive post-market surveillance systems, and establishing specialty-specific XAI clinical practice guidelines. Long-term aspirations for 5+ years encompass achieving regulatory approval for interpretable AI systems in critical applications, establishing a robust evidence base for XAI impact on patient outcomes, developing next-generation interpretable AI architectures, and creating comprehensive international standards for medical AI accountability.

## 8. Limitations

Our systematic review and proposed framework acknowledge several important methodological limitations that may influence the interpretation and generalizability of our findings. The systematic review constraints include the limitation to English-language publications, potentially introducing language bias that may exclude important contributions from non-English speaking researchers and institutions. Heterogeneity in XAI evaluation methodologies across studies limited direct comparisons and required the use of random-effects models, reducing precision in our meta-analytic estimates. Publication bias toward positive results may have inflated reported fidelity scores, as studies demonstrating poor XAI performance may be less likely to be published or may receive less attention in the literature. Additionally, the cross-sectional nature of our analysis cannot establish causality between the XAI method choice and clinical outcomes, limiting our ability to make definitive recommendations about optimal approaches.

Meta-analysis limitations include significant heterogeneity (I^2^ > 75%) that required random-effects models, reducing precision in pooled estimates and highlighting the substantial variation in XAI evaluation approaches across studies. The limited standardization of fidelity measurement protocols across studies created challenges in comparing results and may have introduced systematic bias in our analyses. The retrospective nature of the included studies may not accurately reflect real-world clinical performance, as controlled research environments often differ substantially from routine clinical practice. Variable follow-up periods and outcome definitions across studies further complicated the synthesis of evidence and may have influenced our conclusions about XAI’s effectiveness.

Framework limitations encompass both implementation challenges and generalizability constraints that may affect the practical application of our recommendations. Our three-pillar framework requires significant organizational commitment and may be challenging to implement in resource-constrained settings where technical expertise, financial resources, or institutional support may be limited. Performance trade-offs inherent in prioritizing interpretability may not be acceptable for all clinical applications, particularly in settings where diagnostic accuracy is paramount and small decreases in performance could have significant clinical consequences. Expertise requirements for rule development and validation may not be available in all clinical settings, potentially limiting the feasibility of implementing interpretable-by-design approaches.

Technical limitations include the evolving nature of current metrics for measuring explanation fidelity and clinical utility, which may not capture all the relevant aspects of XAI performance in clinical contexts. Our chosen fidelity measures may not comprehensively represent the complex relationships between explanation quality and clinical decision-making, and limited consensus on appropriate benchmarks for medical XAI evaluation creates challenges in standardizing assessment approaches. The scope of our analysis, focused on three imaging modalities, may limit generalizability to other medical domains where different technical and clinical considerations may apply. Temporal limitations of the current literature may not reflect the rapidly evolving XAI methodologies, and the limited representation of emerging interpretable AI architectures in the existing literature may have influenced our recommendations toward currently available rather than potentially superior future approaches.

## 9. Conclusions

The explainability trap represents a critical challenge for medical AI deployment, with our systematic review and meta-analysis of 67 studies demonstrating that current post hoc explanations misrepresent model reasoning in 55–68% of cases. This comprehensive analysis reveals fundamental limitations in popular XAI methods across medical imaging modalities, with LIME achieving superior fidelity (0.81) compared to SHAP (0.38) and Grad-CAM (0.54), contradicting common assumptions about gradient-based explanations.

Our findings reveal modality-specific patterns demanding tailored XAI approaches rather than one-size-fits-all solutions. The consistent 5–7% AUC performance penalty for interpretable models across all modalities suggests that this trade-off may represent a fundamental architectural limit requiring careful consideration in clinical deployment decisions.

Key Contributions:

1. Empirical Evidence: This is the first comprehensive meta-analysis quantifying XAI method performance across medical imaging modalities.
2. Three-Pillar Framework: This study provides a novel accountability framework distinguishing transparency, interpretability, and explainability.
3. Clinical Validation: We also provide a demonstration of superior clinical outcomes using interpretable alternatives in three major imaging domains.
4. Implementation Guidelines: Practical risk-stratified deployment protocols for healthcare institutions are outlined.

Clinical Implications: Our findings suggest that the medical imaging community must move beyond the false comfort of post hoc explanations toward building genuinely transparent AI systems. The documented performance–interpretability trade-off may be an acceptable exchange for life-critical decisions where understanding is paramount for patient safety.

Immediate Recommendations:

1. Regulatory Action: Implement uncertainty quantification requirements for all clinical XAI systems.
2. Institutional Policy: Establish risk-stratified XAI deployment protocols based on clinical impact.
3. Clinical Practice: Prioritize interpretable models for high-stakes diagnostic decisions.
4. Research Priority: Develop modality-specific XAI evaluation standards and benchmarks.

Future Research Priorities: Critical next steps include prospective clinical trials validating our three-pillar framework, the development of standardized XAI evaluation benchmarks for medical applications, and longitudinal studies assessing XAI impact on patient outcomes and clinical workflow efficiency. The field must also address the urgent need for domain-specific interpretable architectures that minimize the performance–interpretability trade-off.

The stakes of this transition extend beyond academic debate to fundamental questions of patient safety and clinical effectiveness. By embracing a more nuanced understanding of AI accountability that prioritizes transparency and interpretability while treating explainability as a supplementary tool, the medical imaging community can work toward AI systems that genuinely enhance rather than compromise human expertise and patient care.

Only through this commitment to genuine accountability can we realize the transformative potential of artificial intelligence while preserving the trust, understanding, and clinical judgment that form the foundation of effective medical practice. The future of AI in medical imaging depends on our willingness to move beyond the explainability trap toward the more challenging but essential work of building truly accountable AI systems.

## Figures and Tables

**Table 1:** Comparison of explainable AI methods in medical imaging. This table presents the key characteristics, advantages, disadvantages, and clinical limitations of three dominant XAI approaches used in medical imaging applications, based on systematic review findings.

Method	Mechanism	Advantages	Disadvantages	Clinical Gap
SHAP	Feature attribution	Model-agnostic, theoretical foundation	Low fidelity (0.38), computationally expensive	Misleading importance scores
LIME	Local approximation	Interpretable explanations, fast computation	Unstable, poor global fidelity	Inconsistent clinical guidance
Grad-CAM	Gradient-based attention	Visual intuitive, CNN-specific	Coarse resolution, gradient dependence	Limited pathological detail

**Table 2:** Explanation fidelity scores with 95% confidence intervals for three XAI methods across medical imaging modalities. LIME consistently demonstrates superior fidelity across all modalities, while SHAP shows consistently poor performance. Grad-CAM achieves moderate fidelity scores. Color coding indicates performance levels: green (high fidelity ≥ 0.7), yellow (moderate fidelity 0.5–0.69), and red (poor fidelity < 0.5). Study counts (n) represent the number of studies analyzed per modality.

Modality	LIME	SHAP	Grad-CAM
Overall Performance	0.81 (0.78–0.84)	0.38 (0.35–0.41)	0.54 (0.51–0.57)
Radiology (*n* = 23)	0.82 (0.79–0.85)	0.38 (0.34–0.42)	0.55 (0.51–0.59)
Pathology (*n* = 19)	0.81 (0.78–0.84)	0.39 (0.35–0.43)	0.52 (0.48–0.56)
Ophthalmology (*n* = 25)	0.81 (0.77–0.85)	0.37 (0.33–0.41)	0.54 (0.50–0.58)

## Data Availability

The data presented in this study are available in the article and Supplementary Materials. The completed PRISMA checklist, list of excluded studies, and detailed search strategies are available in the Supplementary Materials. Raw data extraction forms and analysis codes are available from the corresponding author upon reasonable request.

## Supplementary Materials

The following supporting information can be downloaded at: https://www.mdpi.com/article/10.3390/bioengineering12080879/s1.
