# Supplementary material for: Beyond Post hoc Explanations: A Comprehensive Framework for Accountable AI in Medical Imaging Through Transparency, Interpretability, and Explainability
Source: Bioengineering (Basel). 2025 Aug 15;12(8):879. doi: 10.3390/bioengineering12080879 (PMC12383817; doi:10.3390/bioengineering12080879)
Supplement: Supplementary file 1 [file bioengineering-12-00879-s001.zip › bioengineering-3755157-supplementary.pdf]

PRISMA 2020 Compliance Statement

Methods Section Addition

PRISMA Compliance: This systematic review and meta-analysis was conducted and reported in accordance with the Preferred Reporting Items for Systematic Reviews and Meta-Analyses (PRISMA) 2020 statement. The completed PRISMA 2020 checklist is provided as Supplementary Material, and the PRISMA flow diagram is included as Figure 1.

Protocol Registration: This systematic review protocol was not prospectively registered. However, all methodological decisions were made a priori and documented before data extraction commenced.

PRISMA 2020 Checklist

Section/Topic	Item #	Checklist Item	Location in Manuscript
**TITLE**			
Title	1	Identify the report as a systematic review	Title page
**ABSTRACT**			
Abstract	2	See PRISMA 2020 for Abstracts checklist	Abstract
**INTRODUCTION**			
Rationale	3	Describe the rationale for the review in the context of existing knowledge	Introduction, page 2-3
Objectives	4	Provide an explicit statement of the objective(s) or question(s) the review addresses	Introduction, page 3
**METHODS**			
Eligibility criteria	5	Specify the inclusion and exclusion criteria for the review and how studies were grouped for the syntheses	Methods, Section 2.2

Information sources	6	Specify all databases, registers, websites, organizations, reference lists and other sources searched or consulted to identify studies	Methods, Section 2.1
Search strategy	7	Present the full search strategies for all databases, registers and websites, including any filters and limits used	Methods, Section 2.1
Selection process	8	Specify the methods used to decide whether a study met the inclusion criteria of the review	Methods, Section 2.3
Data collection process	9	Specify the methods used to collect data from reports	Methods, Section 2.4
Data items	10a	List and define all outcomes for which data were sought	Methods, Section 2.4
Data items	10b	List and define all other variables for which data were sought	Methods, Section 2.4
Study risk of bias assessment	11	Specify the methods used to assess risk of bias in the included studies	Methods, Section 2.5-2.6
Effect measures	12	Specify for each outcome the effect measure(s) used in the synthesis or presentation of results	Methods, Section 2.5-2.7
Synthesis methods	13a	Describe the processes used to decide which studies were eligible for each synthesis	Methods, Section 2.7-2.8
Synthesis methods	13b	Describe any methods required to prepare the data for presentation or synthesis	Methods, Section 2.5-2.7
Synthesis methods	13c	Describe any methods used to tabulate or visually display results of individual studies and syntheses	Methods, Section 2.8

Synthesis methods	13d	Describe any methods used to synthesise results and provide a rationale for the choice(s)	Methods, Section 2.8
Synthesis methods	13e	Describe any methods used to explore possible causes of heterogeneity among study results	Methods, Section 2.8
Synthesis methods	13f	Describe any sensitivity analyses conducted to assess robustness of the synthesised results	Methods, Section 2.8
Reporting bias assessment	14	Describe any methods used to assess risk of bias due to missing results in a synthesis	Methods, Section 2.8
Certainty assessment	15	Describe any methods used to assess certainty (or confidence) in the body of evidence for an outcome	Methods, Section 2.8
**RESULTS**			
Study selection	16a	Describe the results of the search and selection process, from the number of records identified in the search to the number of studies included in the review, ideally using a flow diagram	Results, Section 3; Figure 1
Study selection	16b	Cite studies that might appear to meet the inclusion criteria, but which were excluded, and explain why they were excluded	Supplementary Materials
Study characteristics	17	Cite each included study and present its characteristics	Results, Section 3; Tables
Risk of bias in studies	18	Present assessments of risk of bias for each included study	Results, Section 3.1
Results of individual studies	19	For all outcomes, present, for each study: (a) summary statistics for each	Results, Tables 2-3

		group and (b) an effect estimate and its precision	
Results of syntheses	20a	For each synthesis, briefly summarise the characteristics and risk of bias among contributing studies	Results, Section 3.1
Results of syntheses	20b	Present results of all statistical syntheses conducted	Results, Section 3.1; Figure 2
Results of syntheses	20c	Present results of all investigations of possible causes of heterogeneity among study results	Results, Section 3.1
Results of syntheses	20d	Present results of all sensitivity analyses conducted to assess the robustness of the synthesised results	Results, Section 3.1
Reporting biases	21	Present assessments of risk of bias due to missing results for each synthesis assessed	Results, Section 3.1
Certainty of evidence	22	Present assessments of certainty (or confidence) in the body of evidence for each outcome assessed	Results, Section 3.2
**DISCUSSION**			
Discussion	23a	Provide a general interpretation of the results in the context of other evidence	Discussion, Section 4-5
Discussion	23b	Discuss any limitations of the evidence included in the review	Discussion, Section 8
Discussion	23c	Discuss any limitations of the review processes used	Discussion, Section 8
Discussion	23d	Discuss implications of the results for practice, policy, and future research	Discussion, Section 7
**OTHER INFORMATION**			

Registration and protocol	24a	Provide registration information for the review, including register name and registration number, or state that the review was not registered	Methods, Section 2
Registration and protocol	24b	Indicate where the review protocol can be accessed, or state that a protocol was not prepared	Methods, Section 2
Registration and protocol	24c	Describe and explain any amendments to information provided at registration or in the protocol	N/A - not registered
Support	25	Describe sources of financial or non-financial support for the review, and the role of the funders or sponsors in the review	Funding section
Competing interests	26	Declare any competing interests of review authors	Conflicts of Interest section
Availability of data, code, and other materials	27	Report which of the following are publicly available and where they can be found	Data Availability Statement

---

Abstract Checklist (PRISMA 2020 for Abstracts)

Section/Topic	Item #	Checklist Item	Status
**TITLE**			
Title	1	Identify the report as a systematic review	✓
**BACKGROUND**			
Objectives	2	Provide an explicit statement of the main objective(s) or question(s) the review addresses	✓
**METHODS**			

Eligibility criteria	3	Specify the inclusion and exclusion criteria for the review	✓
Information sources	4	Specify the information sources used to identify studies and the date when each was last searched	✓
Risk of bias	5	Specify the methods used to assess risk of bias in the included studies	✓
Synthesis of results	6	Specify the methods used to present and synthesise results	✓
**RESULTS**			
Included studies	7	Give the total number of included studies and participants and summarize relevant characteristics of studies	✓
Synthesis of results	8	Present results for main outcomes, preferably indicating the number of included studies and participants for each	✓
**DISCUSSION**			
Limitations of evidence	9	Provide a brief summary of the limitations of the evidence included in the review	✓
Interpretation	10	Provide a general interpretation of the results and important implications	✓
**OTHER**			
Funding	11	Specify the primary source of funding for the review	✓
Registration	12	Provide the register name and registration number	N/A - not registered
